# Absence of Regulatory T Cells Causes Phenotypic and Functional Switch in Murine Peritoneal Macrophages

**DOI:** 10.3389/fimmu.2018.02458

**Published:** 2018-10-31

**Authors:** Jelena Skuljec, Adan Chari Jirmo, Anika Habener, Steven R. Talbot, Refik Pul, Ruth Grychtol, Malik Aydin, Christoph Kleinschnitz, Christine Happle, Gesine Hansen

**Affiliations:** ^1^Department of Pediatric Pneumology, Allergology and Neonatology, Hannover Medical School, Hannover, Germany; ^2^Department of Neurology, Essen University Hospital, Essen, Germany; ^3^Biomedical Research in Endstage and Obstructive Lung Disease Hannover (BREATH), Member of the German Center for Lung Research (DZL), Hannover, Germany; ^4^Institute for Laboratory Animal Science and Central Animal Facility, Hannover Medical School, Hannover, Germany; ^5^HELIOS Medical Center Wuppertal, Center for Clinical & Translational Research (CCTR), Faculty of Health, Center for Biomedical Education & Research, Witten/Herdecke University, Wuppertal, Germany

**Keywords:** peritoneal macrophages, small peritoneal macrophages, large peritoneal macrophages, regulatory T cells, scurfy, inflammation, immune regulation, chipcytometry

## Abstract

Tissue macrophages are important components of tissue homeostasis and inflammatory pathologies. In the peritoneal cavity, resident macrophages interact with a variety of immune cells and can exhibit broad range of phenotypes and functions. Forkhead-box-P3 (FOXP3)^+^ regulatory T cells (Tregs) play an indispensable role in maintaining immunological tolerance, yet whether, and how the pathological condition that results from the lack of functional Tregs affects peritoneal macrophages (PM) is largely unknown. We used FOXP3-deficient scurfy (Sf) mice to investigate PM behavior in terms of the missing crosstalk with Tregs. Here, we report that Treg deficiency induced a marked increase in PM numbers, which was reversed after adoptive transfer of CD4^+^ T cells or neutralization of macrophage colony-stimulating factor. *Ex vivo* assays demonstrated a pro-inflammatory state of PM from Sf mice and signs of excessive activation and exhaustion. In-depth immunophenotyping of Sf PM using single-cell chipcytometry and transcriptome analysis revealed upregulation of molecules involved in the initiation of innate and adaptive immune responses. Moreover, upon transfer to non-inflammatory environment or after injection of CD4^+^ T cells, PM from Sf mice reprogramed their functional phenotype, indicating remarkable plasticity. Interestingly, frequencies, and immune polarization of large and small PM subsets were dramatically changed in the FOXP3-deficient mice, suggesting distinct origin and specialized function of these subsets in inflammatory conditions. Our findings demonstrate the significant impact of Tregs in shaping PM identity and dynamics. A better understanding of PM function in the Sf mouse model may have clinical implication for the treatment of immunodysregulation, polyendocrinopathy, enteropathy, X-linked (IPEX) syndrome, and other forms of immune-mediated enteropathies.

## Introduction

Macrophages are tissue sentinels which continuously survey and maintain tissue homeostasis by providing host defense, immune regulation, and wound healing (1, 2). Nevertheless, their uncontrolled activation can result in tissue damage and progression of autoimmune diseases (3, 4), which makes them attractive therapeutic targets (5).

The peritoneal cavity (PerC) is a compartment where macrophages cohabitate with other immune cells. However, interactions and regulation of these cells are poorly understood. It has been indicated that regulatory T cells (Tregs) directly modulate differentiation and function of peritoneal macrophages (PM) (6). In our previous work, we found that the lack of Tregs causes a systemic shift in myeloid lineage commitment and monocyte frequencies in scurfy (Sf) mutant mouse (7), a model for immunodysregulation, polyendocrinopathy, enteropathy, X-linked (IPEX) syndrome. IPEX syndrome is a rare multi-organ autoimmune disorder associated with neonatal onset and early mortality. It is caused by mutations in the gene encoding transcription factor Forkhead-box-P3 (FOXP3) which results in loss of functional Tregs. Despite the fact that severe enteropathy and peritonitis represent common clinical manifestations and are a cause of death in children suffering from IPEX (8, 9), to date there is no data on PM in the particular *in vivo* inflammatory environment caused by the absence of Treg-mediated immune control.

Even though PM represent an extensively studied macrophage population, the existence of two PM subsets in the PerC has only recently been recognized (10). Large peritoneal macrophages (LPM) and small peritoneal macrophages (SPM) display distinct morphologies and phenotypes under steady state conditions (11, 12) and their numbers are altered after inflammatory or infectious stimuli (10, 13–15). However, the knowledge about distribution, origins, functional properties, and plasticity of LPM and SPM in the context of primary systemic immunodeficiencies such as IPEX syndrome or its murine equivalent is still lacking.

In this study, we used FOXP3-deficient Sf mice as an experimental model and identified the pathologic polarization of PM *in vivo* in terms of the missing crosstalk with Tregs. Adoptive transfer of wild type (Wt) CD4^+^ T cells to Sf mice as well as macrophage colony-stimulating factor (M-CSF) neutralization lead to normalization of PM counts. In Sf mice, we found a dramatic shift in ratios and immune signatures of the LPM and SPM. Expression of genes involved in modulation of immune response altered upon CD4^+^ T cell injection and upon transfer of PM to non-inflammatory milieu. Together, here we show that inflammatory conditions resulting from the lack of Tregs have great impact on PM immune functions and plasticity.

## Materials and methods

### Mice

FOXP3^+/−^ heterozygous females (B6.Cg-Foxp3^sf^/J), non-affected inbred males, wild-type donor mice, and congenic CD45.1 mice, all with C57BL/6J genetic background, were originally purchased from The Jackson Laboratory (Bar Harbor, Maine, USA). All mice were housed and bred under specific pathogen-free conditions at the animal facility of Hannover Medical School. Male affected Sf mice and healthy littermate control mice of both genders (Wt) were analyzed at 3 weeks of age. All animal experiments were approved by the local animal welfare committee Lower Saxony State Office for Consumer Protection and Food Safety (LAVES) and performed strictly according to their guidelines.

### Isolation of cells

Peritoneal lavage cells were harvested by flushing the PerC with 3–4 × 1 ml of cold sterile Hank's balanced salt solution (Sigma-Aldrich, St. Louis, Missouri, USA). Cells were centrifuged and counted with Cedex HiRes automated cell analyser (Roche, Basel, Switzerland). If needed, erythrocytes were lysed using in-house made ammonium-chloride-potassium lysing buffer. To determine differential cell counts, cytospins were prepared in CytoSpin 4 Cytocentrifuge (Thermo Fisher Scientific, Waltham, Massachusetts, USA) and stained with May-Grünwald/Giemsa (Merck, Darmstadt, Germany).

### Flow cytometry and fluorescence-activated cell sorting (FACS)

Cells were stained with respective anti-mouse monoclonal antibodies (Supplementary Table S1) for 30 min at 4°C, washed, and resuspended in sterile FACS buffer, containing 0.1% bovine serum albumin in phosphate buffered saline (PBS; Lonza, Basel, Switzerland). A 15 min long Fcγ receptor blocking step (unlabelled CD16/32, clone 2.4G2; BD Biosciences, Franklin Lakes, New Jersey, USA) preceded all stainings. Data were acquired on a FACSCantoII (BD Biosciences) and analyzed using FlowJo software V10 (FlowJo LLC, Ashland, Oregon, USA). Cells were sorted by FACSAria Fusion (Becton-Dickinson) at Research Facility Cell Sorting of Hannover Medical School. Apoptosis was assessed with FITC Annexin V Apoptosis Detection Kit (BD Biosciences).

### Gene and protein expression analysis

Total cellular RNA was extracted using the RNeasy Plus Mini or Micro Kit (Qiagen, Venlo, Netherlands) and reversely transcribed with the High-Capacity cDNA Reverse Transcription Kit (Applied Biosystems, Foster City, California, USA). Quantitative PCR was performed with 90 ng RNA in a 7500 Fast Real-Time PCR System (Applied Biosystems). Primers (Supplementary Table S2) and TaqMan Universal Master Mix II were purchased from Applied Biosystems. Expression of genes was relativized to the house-keeping gene using 2^−ΔCt^ algorithm (16). Chemokine and cytokine levels were measured in undiluted peritoneal fluid by bead-based immunoassays (Mouse chemokine-6plex, CXCL1/KC-simplex and Th1/Th2/Th17/Th22-13plex FlowCytomix, eBioscience, San Diego, California, USA).

### Chipcytometry

Iterative chip-based imaging cytometry has been performed as described previously (17, 18) (http://www.zellkraftwerk.com). Cells were fixed onto microfluidic ZellSafe™ chips (Zellkraftwerk GmbH, Leipzig, Germany) and the expression of surface markers was measured microscopically on the same cells in a cyclic staining–imaging–bleaching process (Axio Imager M1; Carl Zeiss, Jena, Germany) with original magnification x160. All monoclonal antibodies used were PE-conjugated (Supplementary Table S1), diluted in PBS, and incubated with cells 10–15 min at room temperature. Imaging and data analyses were performed using ZellScanner ONE software (Zellkraftwerk). After excluding autofluorescence, cell-individual MFI values for all scanned markers were analyzed with GraphPad Prism (GraphPad Software, Inc., San Diego, California, USA). Hierarchical clustering was used to generate heatmaps and linear discriminant analysis with the SAS JMP 12 software (SAS Institute, Cary, North Carolina, USA). For tissue chipcytometry, cuts were prepared from dissected *omentum majus* incubated over-night in 4% formalin and mounted on ZellSafe™ chip for tissue sections. Cells were stained as described above and nuclei were counterstained with Hoechst 33342 dye. Positive fluorescence signals were subtracted from respective background signals and presented in different colors. Overlay pictures were created with Adobe Photoshop (Adobe Systems, San Jose, California, USA), as published previously (19). Original chipcytometry pictures of *omentum majus* are shown in Supplementary Figure S6.

### Neutralization of M-CSF *in vivo*

One-week-old pups were intraperitoneally (i.p.) injected with anti-murine M-CSF antibody (20 μg/g of body weight; clone 5A1, Bio X Cell, West Lebanon, New Hampshire, USA) every third day, receiving 6 injections in total. In parallel, control mice were injected with equal amount of isotype control antibody (clone: HRPN, Bio X Cell).

### *Ex vivo* assays

Peritoneal exudates (PE) cells were isolated and plated in RPMI 1640 medium supplemented with 10% fetal bovine serum and 1% penicillin/streptomycin (RPMI+; all from Biochrom AG, Berlin, Germany). After 2 h of incubation, purified PM were washed, detached by 2mM PBS/EDTA, counted and plated in RPMI+ medium (2 × 10^5^ cells/96 well). Secretion of NO by PM was measured in supernatants after over-night stimulation with 100 ng/ml lipopolysaccharide LPS (L2654; Sigma-Aldrich) in Griess assay, as described previously (20).

For the assessment of metabolic activation, PM were purified from freshly isolated PE with EasySep™ Mouse Monocyte Isolation Kit (Stemcell Technologies, Vancouver, Canada). Cells were plated (10^5^ in 100 μl RPMI+ medium) and ATP amount was determined by CellTiter-Glo® and Glomax luminometer (Promega, Madison, Wisconsin, USA).

Autophagy in PM was measured in freshly isolated PE by flow cytometry according to the kit manual (Cyto-ID® autophagy detection kit, Enzo Life Sciences, Farmingdale, New York, USA) in parallel with staining of surface markers.

### *In vivo* proliferation and phagocytosis

Sterile bromodeoxyuridine (BrdU) solution (1 mg in PBS, 100 μl/mouse) was i.p. injected. After 4 h, cells were isolated, counted, stained for BrdU (FITC BrdU Flow Kit from BD Biosciences) and surface markers, and analyzed by flow cytometry.

Sterile suspension of Fluoresbrite® yellow-green microspheres (1 μm in diameter, Polysciences, Inc., Warrington, Pennsylvania, USA) in PBS (1%) was injected i.p. (100 μl /mouse). After 2 h, PE cells were collected, counted, and stained for surface markers. Phagocytosis rate in PM was assessed by flow cytometry, as previously described (21).

### Adoptive cell transfer

PE cells were harvested from either CD45.1 Wt or CD45.2 Sf mice, stained, and FACS sorted in sterile conditions. FSC^high^SSC^high^CD3^−^CD19^−^Gr-1^−^CD11c^−^SiglecF^−^ PM were injected i.p. to either CD45.2 Sf or CD45.1 Wt mice, respectively (~10^6^ cells in sterile PBS, 150 μm/mouse). PE cells were collected 36 h later. Transferred PM were distinguished by CD45.1/CD45.2 followed by CD11b^+^F4/80^+^ staining and FACS sorted. Average cell recovery upon transfer was 7.4% of total injected cells.

For adoptive T cell transfer, single-cell suspensions were obtained from spleens and lymph nodes of 10-12 weeks old C57BL/6 donor mice by meshing through a 70 μm cell strainer (BD Biosciences), followed by lysis of erythrocytes. T cells were isolated with “Mouse CD4^+^ T Cell Isolation Kit II” by MACS cell separation (Miltenyi Biotec, Bergisch Gladbach, Germany), resuspended in 100 μl of sterile PBS (5 × 10^6^ cells), and injected i.p. into 7 days old Sf and littermate control pups. Mice were sacrificed 2 weeks post-injection, PE was isolated and CD115^+^CD11b^+^F4/80^+^ PM and peritoneal CD4^+^ cells were FACS sorted.

### Microarray experiments (single-color mode)

The microarray utilized in this study represents a refined version of the Whole Mouse Genome Oligo Microarray 4 × 44K v2 (Design ID 026655, Agilent Technologies, Santa Clara, California, USA). Microarray design was created at Agilent's eArray portal using a 4 × 180K design format for mRNA expression. 40 ng of total RNA were used to prepare aminoallyl-UTP-modified (aaUTP) cRNA (Amino Allyl MessageAmp™ II Kit, Thermo Fisher Scientific). Prior to the reverse transcription, 1 μl of Agilent's One-Color spike-in Kit stock solution (1:100000) was added to each analyzed sample. The labeling of aaUTP-cRNA was performed with Alexa Fluor 555 Reactive Dye (Thermo Fisher Scientific). cRNA fragmentation, hybridization and washing steps were carried-out as recommended in the “One-Color Microarray-Based Gene Expression Analysis Protocol V5.7.” Slides were scanned using the Agilent Micro Array Scanner G2565CA. Data extraction was performed with the “Feature Extraction Software V10.7.3.1” (protocol file “GE1_107_Sep09.xml”). Average expression intensities were row normalized for each experiment and from this heatmaps were constructed. Rows were clustered using the complete linkage method which finds similar clusters. All calculations were performed in the R software using the libraries “pheatmap” and “gplots.”

### Statistical analysis

Unpaired two-tailed Student's *t*-test or one-way ANOVA test with Bonferroni's multiple comparison post-test was performed (95% confidence interval) using GraphPad Prism. Data are shown as mean ± SEM. Differences were considered significant at *P* < 0.05 (^*^), *P* < 0.01 (^**^), and *P* < 0.001 (^***^). The number of single mice or pooled samples per experimental group is indicated with “n” in each corresponding figure legend.

## Results

### PM numbers and pro-inflammatory cytokine levels are increased in the perC of sf mice

**Figure 1:**
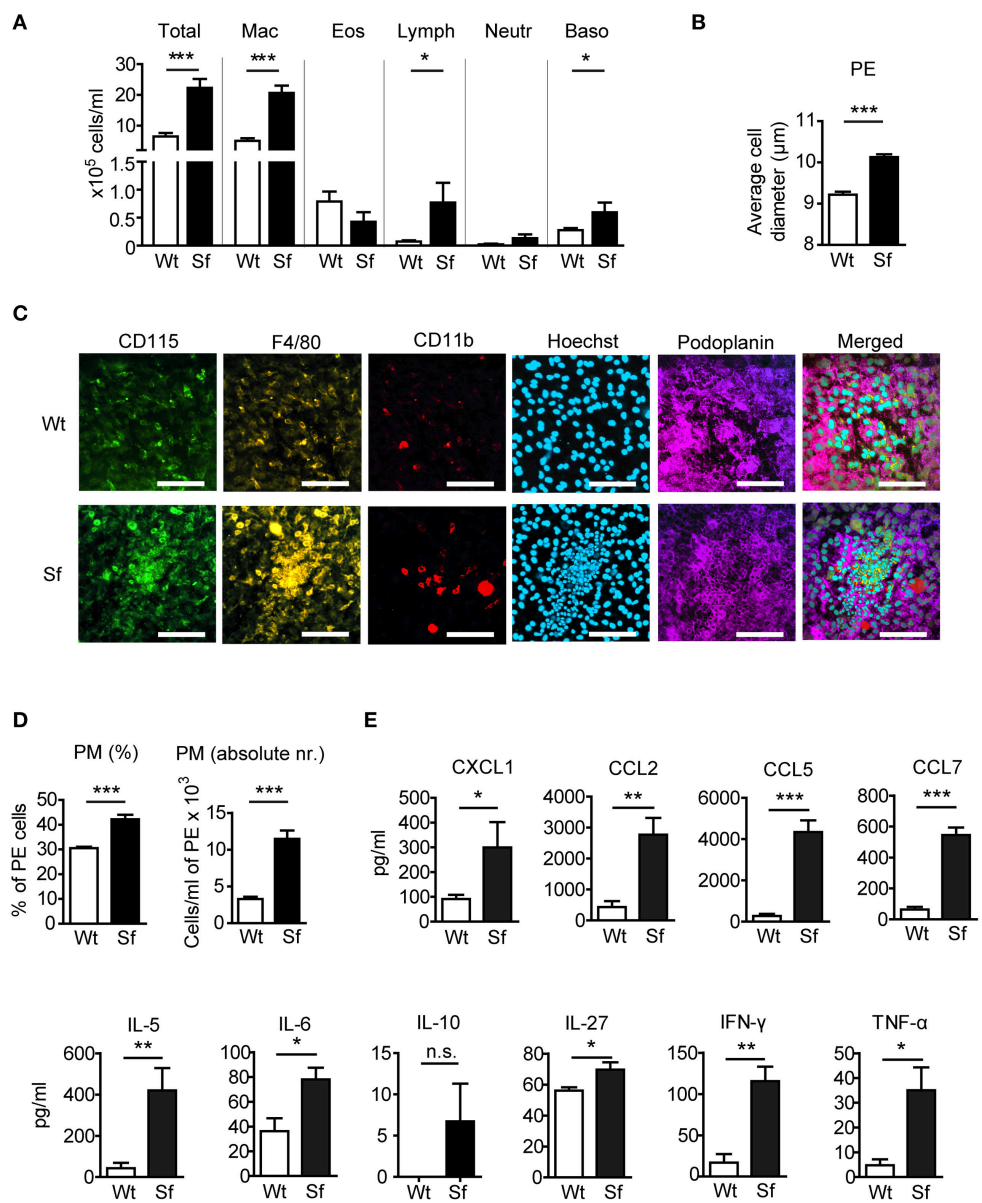
Mice that lack regulatory T cells have elevated peritoneal and macrophage counts and cytokine/chemokine levels**. (A)** Total and differential counts of peritoneal exudate (PE) cells isolated from scurfy (Sf) mice or littermate controls (Wt), fixed with cytospin centrifuge, and stained with May-Grünwald/Giemsa (*n* > 15 per group). **(B)** Average cell diameter in PE determined by automated cell analyser (*n* = 9–10). **(C)** Chipcytometry stainings of *omentum majus* fixed on ZellSafe™ chips; scale bar = 100 μm. **(D)** Percentages (*n* = 8–9) total cell numbers (*n* = 5–6) of CD115^+^CD11b^+^F4/80^+^ peritoneal macrophages (PM) determined by flow cytometry and automated cell analyser. **(E)** Chemokine and cytokine levels in peritoneal fluid, measured by bead-based multiplex immunoassay (*n* = 5 samples, each pooled from 2–3 mice). Statistical analyses were performed using unpaired Student's *t*-test, *p < 0.05, **p < 0.01, ***p < 0.001. n.s., not significant; Mac, macrophages; Eos, eosinophils; Lymph, lymphocytes; Neutr, neutrophils; Baso, basophils.

To examine the impact of Treg deficiency on the immune state of the peritoneal compartment, we analyzed PE from FOXP3 deficient Sf mice and compared them to their littermate controls. In Sf mice we found a significantly higher number of peritoneal cells comprising mainly of PM (Figure 1A). The average cell diameter in the PE of Sf mice was greater than in healthy mice, probably reflecting the high frequency of enlarged activated macrophages (Figure 1B). Immunohistological examination of the *omentum majus* sections using chipcytometry revealed aggregates of inflammatory immune cells in Sf mice that contained CD115^+^F4/80^+^CD11b^+^ PM (Figure 1C). The increased percentage and absolute number of PM in the PerC of Sf mice were further corroborated by flow cytometry (Figure 1D). PM were identified by the expression of CD115, CD11b, and F4/80 markers after exclusion of T and B lymphocytes, dendritic cells, infiltrating monocytes, and granulocytes, using a cocktail of antibodies against CD3, CD19, Gr-1 (Ly6G/Ly6C), Siglec F, and CD11c (Supplementary Table S1).

Analysis of chemokine and cytokine levels in the PerC revealed significant increase in secretion of chemokines CXCL1, CCL2, CCL5, and CCL7, and pro-inflammatory cytokines IL-5, -6, -27, IFN-γ, and TNF-α in Sf mice, whereas IL-10 levels were not significantly different between the two groups (Figure 1E).

### PM in Sf mice exhibit pro-inflammatory polarization

**Figure 2:**
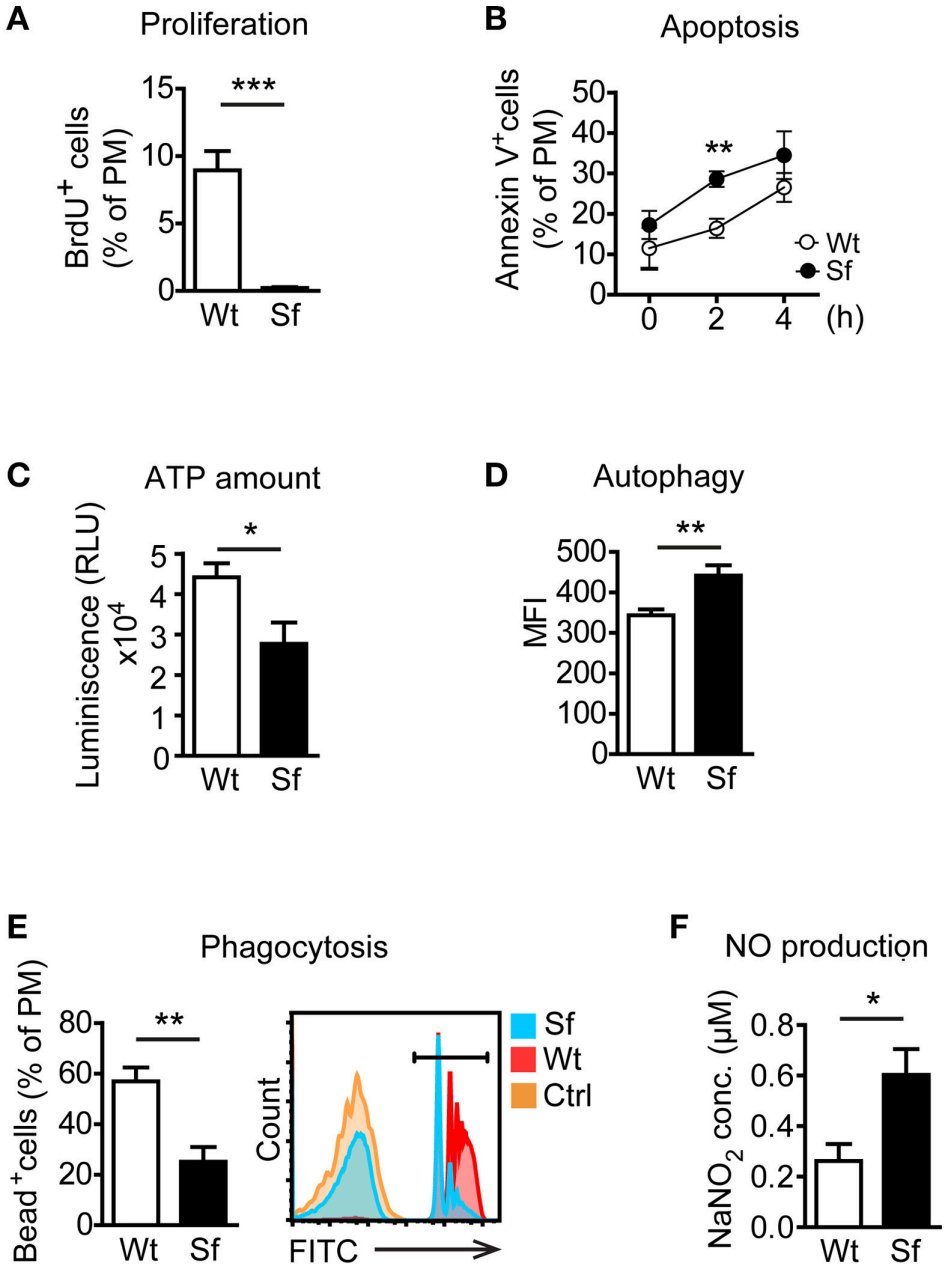
Peritoneal macrophages in scurfy mice acquire state of activation and exhaustion. **(A)** *In vivo* proliferation (*n* = 4, one representative experiment out of three) and (**B)** apoptosis (Annexin V staining; *n* = 9–13 per time-point) of peritoneal macrophages (PM) in control (Wt) and scurfy (Sf) mice measured by flow cytometry. **(C)** ATP amount in purified PM quantified by CellTiter-Glo (*n* = 3–5). Flow cytometry analysis of **(D)** autophagy (*n* = 4–5, one representative experiment out of two) and **(E)** phagocytosis *in vivo* (*n* = 5) in Wt and Sf PM. **(F)** Nitrogen oxide (NO) secretion by purified PM measured by Griess assay (*n* = 4–8). Statistical analyses were performed using unpaired Student's *t*-test, *p < 0.05, **p < 0.01, ***p < 0.001.

Next, we examined metabolic and functional properties of PM deriving from a Treg-deprived inflammatory environment. With i.p. BrdU injection we labeled cells generated by proliferation. A considerable extent of BrdU incorporation by Wt PM (Figure 2A) corroborated proliferation as a mechanism of the PM renewal in healthy conditions (22). In contrast, Sf PM showed remarkably lower BrdU incorporation *in vivo*, indicating that recruitment of PM precursors or tissue macrophages from different location rather than proliferation accounts for their increased number in Sf mice. PM from Sf mice showed a higher apoptosis rate and a lower cell energy level than their Wt counterparts (Figures 2B,C). Furthermore, Sf PM demonstrated increased autophagic activity (Figure 2D), reduced phagocytosis rate (Figure 2E) and an elevated production of nitric oxide (NO; Figure 2F) when compared to the PM from Wt littermates, indicating high activation and stress condition of these cells. PE cells isolated from Sf mice displayed poor colony formation and contained very low amount of Lin^−^c-kit^+^Sca-1^−^ myeloid progenitors and lineage marker negative hematopoietic stem and progenitor cells (not shown).

### Immune activation of Sf PM is attenuated upon their transfer to non-inflammatory environment

**Figure 3:**
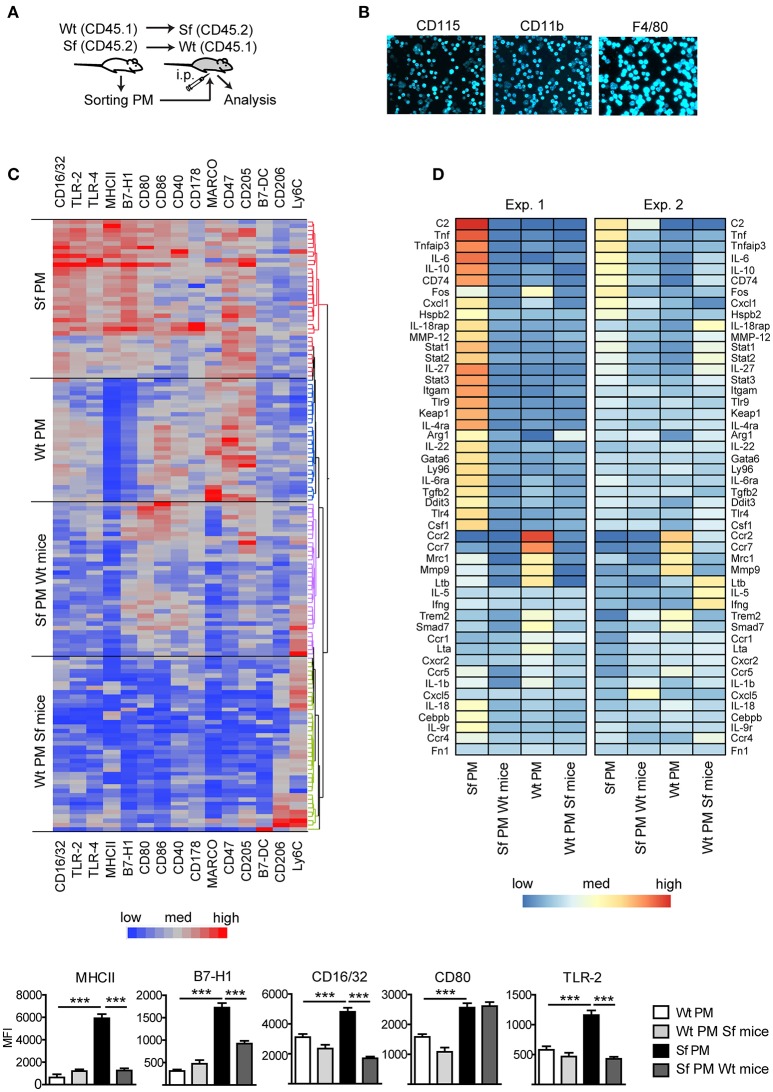
Immunophenotyping by single-cell chipcytometry and transcriptomics reveal plasticity of peritoneal macrophage activation profiles**. (A)** Schematic outline of the experimental protocol, **(B)** exemplary surface marker stainings of the cells fixed on chip, and **(C)** immunophenotyping of scurfy (Sf) and unaffected (Wt) peritoneal macrophages (PM) from the untreated mice and after their transfer to Wt or Sf peritoneal cavity (“Sf PM Wt mice” and “Wt PM Sf mice”), respectively, performed by single-cell chipcytometry. Data are depicted as mean fluorescent intensities (MFI) for each surface marker expressed by single cells and shown as heatmaps (middle) and bar graphs (bottom; n~60 cells per group, pooled from more than 8 donor mice). **(D)** Microarray-based transcriptome analysis of gene expression in PM from untreated Sf and Wt mice and after their intraperitoneal (i.p.) transfer to Wt or Sf mice, respectively (“Sf PM Wt mice” and “Wt PM Sf mice”; two independent experiments, each experimental group contains pooled cells from more than 5 mice). Statistical analysis was performed using one-way ANOVA, ***p < 0.001.

Subsequently, we measured in PM expression of molecules involved in initiation and exacerbation of adaptive immune response, antigen uptake and processing, activation of innate immune response, homing/trafficking, apoptosis, limitation and resolution of inflammation, and tissue dependent phenotype. In order to determine the contribution of environmental signals on PM immune profiles, we additionally isolated PM from CD45.1 Wt mice or CD45.2 Sf mice by FACS and transferred i.p. into Sf mice (CD45.2) or CD45.1 Wt recipient mice, respectively (Figure 3A). After 36 h, PM were FACS sorted and analyzed by single-cell chipcytometry (Figures 3B,C) and transcriptomics (Figure 3D). PM from Sf and Wt mice harbored distinct expression profiles of immune markers (Figures 3C,D). Upon transfer to Wt mice, the engrafted Sf PM retained high levels of CD115, CD11b, and F4/80 surface expression (Figure 3B). However, expression of surface markers and genes involved in activation of innate inflammatory response, cellular stress response, and regulation of adaptive immune reaction such as antigen presentation, binding of antibody-antigen immune complexes, regulation of T cell activation, and cytokine cascade in Sf PM were downregulated to levels equivalent to PM in healthy mice (Figures 3C,D, Supplementary Figure S1). In contrast, Wt PM transplanted to the PerC of Sf mice did not remarkably upregulate genes that were highly expressed in Sf PM, suggesting that 36 h might be too short time for the pro-inflammatory priming in the absence of Tregs. Nevertheless, Wt PM downregulated expression of genes such as *Ccr2, Ccr7, Mrc1, Mmp9 Trem2, and Smad7* to levels similar to those of host Sf PM (Figure 3D). This transfer experiment reveals the high extent of functional plasticity of PM and reinforces the role of tissue microenvironment in shaping their functional identity.

### Alterations in number and inflammatory phenotype of PM in Sf mice are restored upon adoptive transfer of Wt CD4^+^ cells

**Figure 4:**
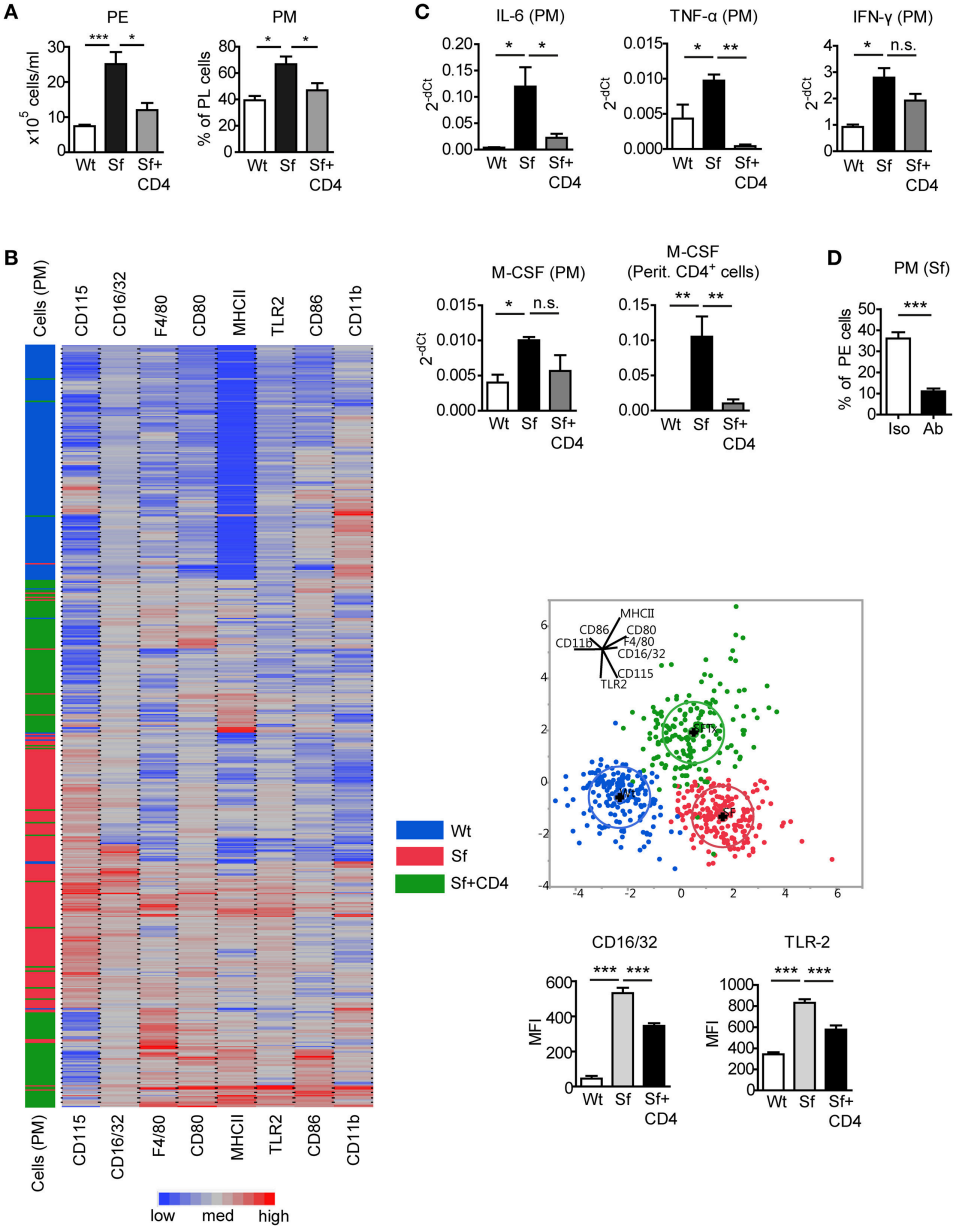
Injection of Wt CD4^+^ T cells to Sf mice restores PM number and inflammatory cytokine expression. **(A)** Total peritoneal exudate (PE) cells and percentages of peritoneal macrophages (PM) in controls (Wt), scurfy mice (Sf), and adoptively transferred Sf mice with Wt CD4^+^ cells (Sf+CD4), analyzed by automated cell counter and flow cytometry, respectively (*n* ≥ 5). **(B)** Heatmap, linear discriminant analysis, and bar graphs of cell surface markers measured by chipcytometry on gated CD115^+^CD11b^+^F4/80^+^ PM (*n* = 200 cells per group, pooled from more than 3 mice). **(C)** Impact of adoptive Wt CD4^+^ T cell transfer on cytokine mRNA expression by PM and peritoneal CD4^+^ T cells, measured by qPCR (*n* = 3–4). **(D)** Percentages of PM in PE of Sf mice after *in vivo* treatment with anti-M-CSF antibody (Ab) or isotype (Iso) control (*n* = 5 mice, data are pooled from two independent experiments). Statistical analyses were performed using one-way ANOVA (A-C) or unpaired Student's *t*-test (D), *p < 0.05, **p < 0.01, ***p < 0.001.

To assess the impact of Tregs on the altered PM counts and polarization in Sf mice, CD4^+^ T cells containing Tregs were isolated from healthy CD45.1 congenic Wt mice and injected intraperitoneally (i.p.) into Sf pups. CD4^+^ T cell transfer normalized total PE cell number and PM frequency in Sf recipient mice (Figure 4A). The cellular composition in the PerC of the control Wt recipient mice was not influenced by the treatment (not shown). Single-cell immunophenotyping of surface markers using high dimensional analysis approach such as linear discriminant analysis revealed that PM from treated Sf mice cluster between two PM populations, isolated from Sf mice and unaffected Wt controls, confirming the regulatory effect of Tregs on PM phenotype (Figure 4B). Furthermore, we found normalization in expression profile of pro-inflammatory cytokines IL-6 and TNF-α in PM isolated from Sf mice treated with CD4^+^ T cells (Figure 4C). Interestingly, treatment with Wt CD4^+^ T cells did not significantly reduce higher expression level of M-CSF mRNA in Sf PM, whereas the injected cells were highly efficient in reducing M-CSF levels in the peritoneal T cells from Sf mice (Figure 4C). In line with this, we previously reported that activated T cells in the bone marrow of Sf mice display high production of M-CSF, which was restored upon CD4^+^ cell transfer (7). Next, we aimed to find out whether M-CSF drives the increased PM generation in Sf mice. Indeed, *in vivo* treatment with anti-M-CSF antibody normalized PM counts in Sf mice, suggesting that the expansion of PM in the absence of Tregs might be dependent on this growth factor (Figure 4D). Of note, anti-M-CSF antibody treatment reduced PM numbers also in Wt mice (Supplementary Figure S2), confirming the key role of M-CSF in controlling PM development in steady state.

### Quantity, origin, and effector function of PM subsets are differently affected by lack of tregs

**Figure 5:**
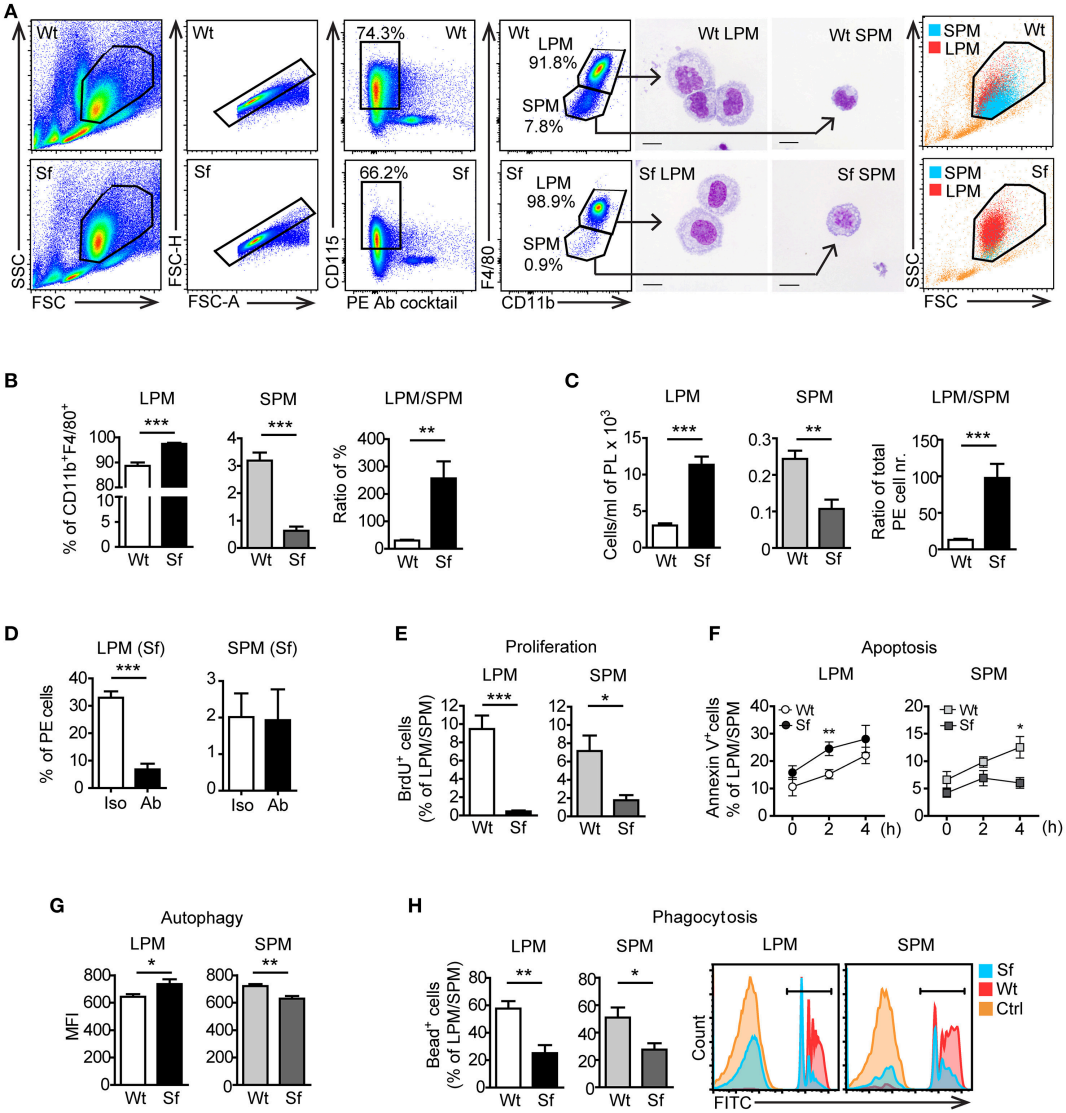
Peritoneal macrophage subsets and their effector functions are skewed due to the absence of regulatory T cells. **(A)** Gating strategy used to identify large peritoneal macrophages (LPM) and small peritoneal macrophages (SPM) by flow cytometry. Cells were FACS sorted and stained with May-Grünwald/Giemsa. Data shown are representative from >10 experiments. **(B)** Percentages (*n* = 8) and **(C)** total cell numbers (*n* = 5–6) of CD115^+^CD11b^high^F4/80^high^ LPM and CD115^+^CD11b^int^F4/80^int^ SPM in the peritoneal exudate of scurfy (Sf) and littermate control (Wt) mice determined by flow cytometry. **(D)** Percentages of LPM and SPM in Sf mice within PE after injection of anti-M-CSF antibody (Ab) or isotype (Iso) control antibody (*n* = 5 mice, data are pooled from two independent experiments). Flow cytometry analyses of **(E)** *in vivo* proliferation (*n* = 4, one representative experiment out of three), **(F)** apoptosis (Annexin V staining; *n* = 8–11 per time-point), **(G)** autophagy (*n* = 5-6), and **(H)** phagocytosis rate *in vivo* (*n* = 5) of LPM and SPM in Sf and Wt mice. Statistical analyses were performed using unpaired Student's *t*-test, *p < 0.05, **p < 0.01, ***p < 0.001.

According to previously published gating strategies (10, 15), we identified CD115^+^CD11b^high^F4/80^high^ LPM and CD115^+^CD11b^int^F4/80^int^ SPM population in the PE after excluding cell aggregates and other peritoneal immune cells using antibody cocktail that binds CD3, CD19, Gr-1 (Ly6G/Ly6C), Siglec F, and CD11c (Figure 5A, Supplementary Table S1). The size, morphology, and purity of the two PM subsets were confirmed by FSC/SSC parameters and by microscopic analysis of the FACS sorted cells (Figure 5A). Sf mice contained significantly higher percentage and absolute number of LPM than Wt controls, whereas SPM population in Sf mice was present in surprisingly low amount (Figures 5A–C). In contrast to SPM from Wt mice and LPM from both Wt and Sf mice, quantity of Sf SPM was not affected by M-CSF neutralization (Figure 5D, Supplementary Figure S2), suggesting disparate developmental pathways governed by additional growth factor(s). Based on the minor BrdU incorporation *in situ*, both LPM and SPM in the PerC of Sf mice seem to have abated proliferation rate (Figure 5E). Sf LPM demonstrated significantly higher apoptotic and autophagic activity than their Wt counterparts, whereas the same processes were significantly diminished in Sf SPM when compared to Wt SPM (Figures 5F,G). Next, we evaluated the *in situ* phagocytic capacity of Wt and Sf PM subsets. Remarkably lower percentage of fluorescent bead-ingesting cells (Figure 5H) and amount of engulfed beads per cell (not shown) reflect poor phagocytic activity in the PerC of Sf mice. Overall, these data reveal dramatic differences in the way that LMP and SPM react on the absence of Treg dependent immune control.

### The expression pattern of immunologic markers sequentially changes as LPM and SPM are exposed to different microenvironment

**Figure 6:**
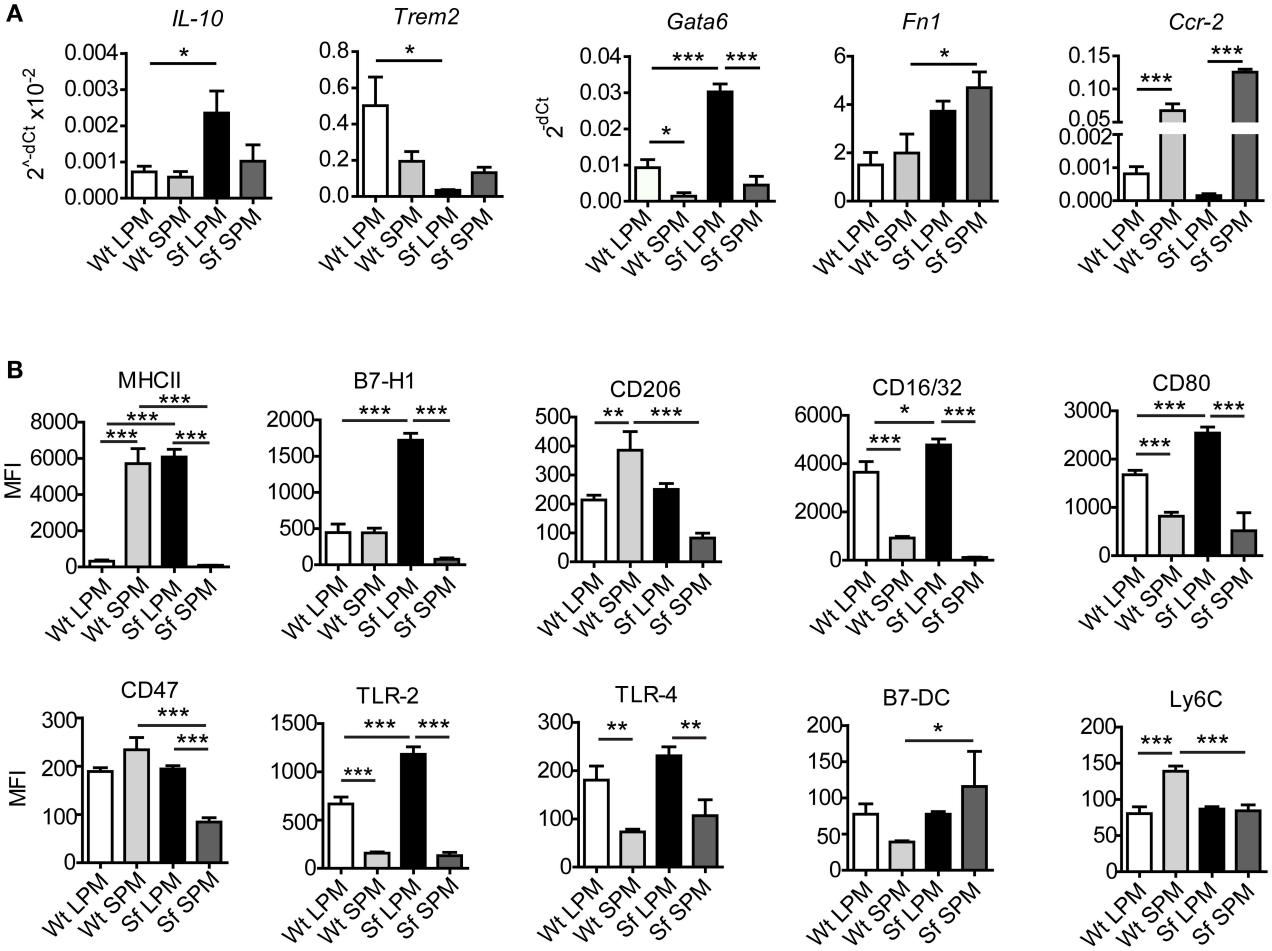
Large and small peritoneal macrophages have distinct immune profiles in steady-state and inflammatory conditions. **(A)** Quantitative PCR analysis of gene expression in large and small peritoneal macrophages (LPM and SPM), isolated from scurfy (Sf) and control (Wt) mice (*n* = 4–7 samples per group, each sample contains pooled cells from 3 to 7 mice). **(B)** Expression pattern of cell surface markers involved in immune response in LPM and SPM analyzed by single-cell chipcytometry. Data are depicted as mean fluorescent intensities (MFI) for each surface marker expressed by single cells (*n* = 28–62 cells per experimental group, each sample contains pooled cells from more than 8 donor mice).

Our next aim was to characterize in-depth the behavior of LPM and SPM in healthy and autoimmune conditions. Quantitative PCR and single-cell chipcytometry analyses revealed that the characteristic expression of a series of genes and surface markers clearly distinguishes LPM from SPM in Wt mice, which was to a great extent, but dissimilarly affected by the inflammatory settings in Sf mice (Figures 6A,B, Supplementary Figures S3, S4). When compared to healthy controls, LPM from Sf mice showed significant upregulation of *Il10, Gata6, Fn1, Cxcl1, Tnfa, Ifng, iNos*, MHCII, B7-H1, CD16/32, CD80, and TLR2, as well as downregulation of *Trem2* expression. In contrast, SPM from Sf mice were characterized by *Ccr2* and B7-DC upregulation along with MHCII, CD206, CD47, and Ly6C downregulation when compared to Wt SPM (Figures 6A,B).

**Figure 7:**
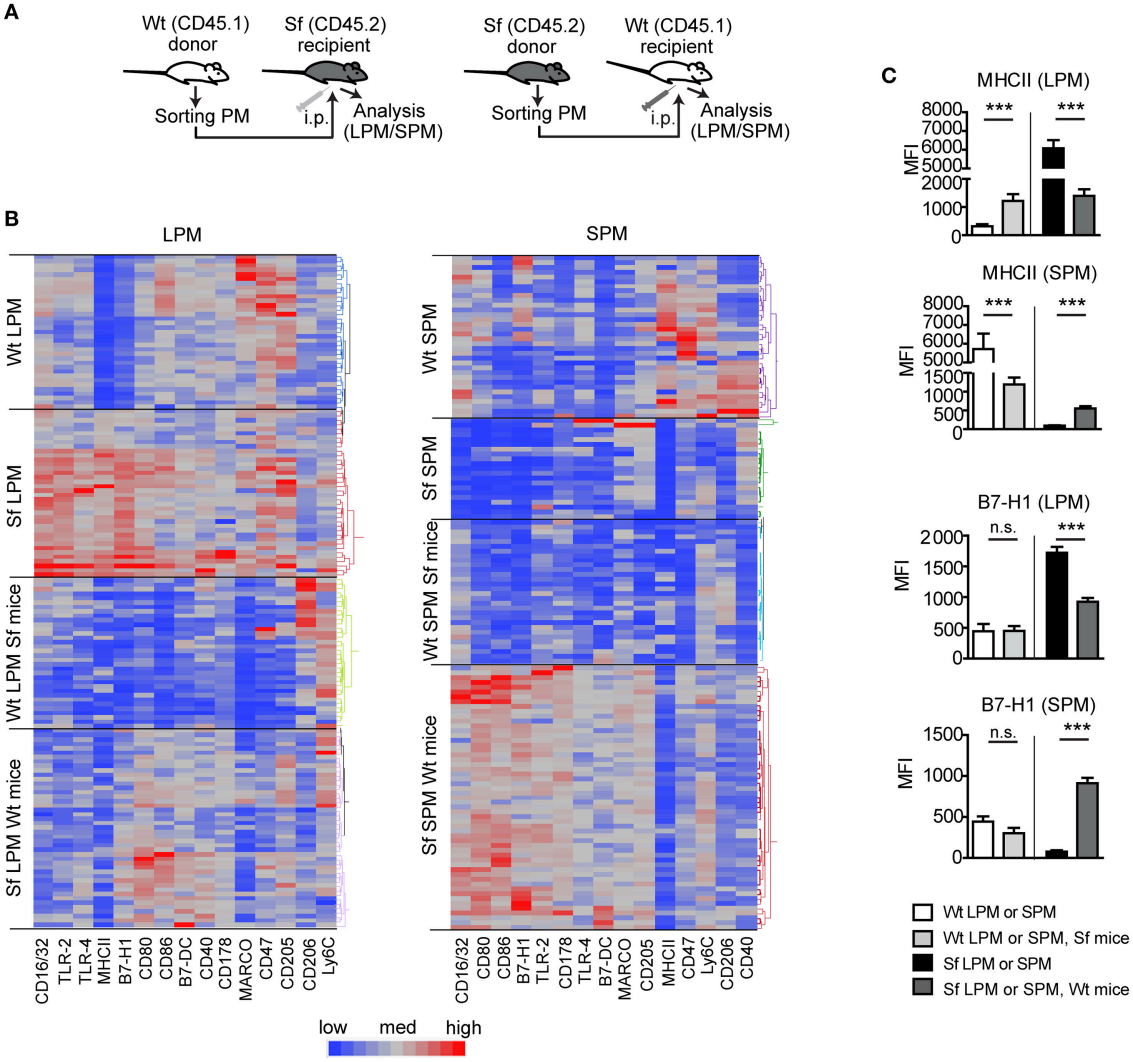
Microenviroment shapes immune signatures of large and small peritoneal macrophages. **(A)** Schematic outline of the experimental protocol and **(B,C)** immunophenotyping by single-cell chipcytometry of control (Wt) and scurfy (Sf) large and small peritoneal macrophages (LPM and SPM), isolated from either non-treated mice or following PM transfer to Sf and Wt mice, respectively. Data are depicted as MFI for each surface marker expressed by single cells and shown as heatmap **(B)** or bar graphs **(C)**. *n* = 28–62 cells per experimental group, each sample contains pooled cells from more than 8 donor mice. Statistical analyses were performed using one-way ANOVA, ***p < 0.001.

We further performed PM transfer from Sf to Wt mice and vice versa and examined to which extent microenvironment controls the functional polarization of the PM subsets (Figure 7A). Thirty-six hours after the interchange in inflammatory conditions we could detect remarkable alterations in the LPM and SPM immune marker patterns (Figures 7B,C, Supplementary Figure S5). The bimodal expression of MHCII on both cell types exhibited the most striking shift upon the transfer in both directions, demonstrating that inflammatory milieu orchestrates prompt activation/resolution of the PM-dependent regulation of adaptive immune reaction. Likewise, the high/low expression of CD16/32 and immunosuppressive marker B7-H1 on LPM/SPM in Sf mice was attenuated/increased when transferred to PerC of Wt mice, illustrating strong environmental influence on phenotypic switch of the PM subsets (Figures 7B,C and Supplementary Figure S5). The expression of some other markers like Ly6C or CD205 was not considerably affected by the environmental change (Supplementary Figure S5). In summary, upon transfer to non-inflammatory environment, Sf LPM promptly downregulated expression of inflammatory markers, whereas Sf SPM turned their phenotype in different direction, suggesting that functional polarization of Sf PM subsets appears to be dictated by the environment, rather than being cell-intrinsic.

## Discussion

It is well established that macrophages play important but complex roles in the regulation of autoimmune diseases and therefore macrophage polarization in the context of inflammation is a topic of great interest (3, 4). Several studies have previously provided a potential link between macrophage function and Treg-mediated immune control (6, 23–25). However, whether and how the pathological condition that results from lack of Tregs affects macrophage function *in vivo* remains unresolved. Here, we used FOXP3-deficient Sf mice to systematically examine dynamics, activation profiles, and plasticity of PM.

We detected PM aggregates in the peritoneum-associated adipose tissue *omentum majus* and a considerable increase of PM counts in the PerC of Sf mice. Under steady state conditions, PM maintain by self-renewal independently of hematopoiesis (26), whereas studies in zymosan induced peritonitis model indicated that the proliferation together with bone marrow-derived monocyte recruitment provides a mechanism for expansion of PM during inflammation (22, 27). We labeled proliferating cells with synthetic nucleoside and detected surprisingly low contribution of local cell division to the enlarged PM population in our model. Next, we checked whether peritoneal extramedullary hematopoiesis (28) represents means for PM abundance and found that hematopoietic stem and progenitor cells were nearly absent in the PerC of Sf mice. Therefore, we conclude that PM in the absence of Tregs originate mainly from actively recruited circulating monocytes and/or tissue macrophages invading via a non-vascular route (29). High levels of chemokines that we detected in the PerC of Sf mice probably contribute to the monocyte/macrophage recruitment. Given that M-CSF represents an important stimulus for the myelopoiesis and differentiation of monocytes/macrophages (30) and that it is highly expressed in Sf mice (7), we neutralized this cytokine and observed a marked reduction in PM number. In our previous study, we noted that the increased proportions of myeloid progenitors and monocyte subsets in the bone marrow of Sf mice also normalized after treatment with anti-M-CSF antibody (7). The combination of these findings places M-CSF as an important mediator in the regulation of monocyte and macrophage counts in the absence of Tregs. Likewise, adoptively transferred CD4^+^ T cells containing FOXP3^+^ Tregs efficiently restored elevated total cell numbers in the PE and PM frequencies. In addition, injected cells reduced high expression of pro-inflammatory citokines and surface markers in Sf PM, as well as high M-CSF mRNA expression in peritoneal CD4^+^ cells. This, together with the fact that FOXP3 is not expressed by PM (unpublished observation), indicates that the recovery of a functional Treg compartment limits the peritoneal inflammation in experimental model of IPEX syndrome via functional reprogramming of PM and peritoneal T cells.

Here we show that the absence of functional Tregs is associated with increased levels of peritoneal pro-inflammatory cytokines which presumably derive from activated PM and further stimulate their activation in a positive-feedback loop manner (31). Sf PM displayed higher NO production and lower phagocytic activity, indicating their pro-inflammatory state, whereas increase in apoptotic and autophagy activity along with low cellular energy levels demonstrates signs of excessive activation and exhaustion. Taken together, these results reveal that PM homeostasis directly depends on the Treg presence, which goes in line with recent observations that Tregs actively influence macrophages (6, 23). However, the revelation of exact mechanisms underlying Treg-mediated regulation requires further studies.

Single-cell chipcytometry and transcriptome analysis also revealed pro-inflammatory polarization of Sf PM, reflected in upregulation of molecules responsible for T cell priming and initiation of innate and adaptive immune responses. It is therefore extremely likely that, in the absence of Tregs, PM aggravate inflammatory cascades thereby contributing to autoimmune pathogenesis. However, besides playing a role in tissue destruction (3, 4), macrophages are able to promote immune tolerance by direct induction of Tregs and suppression of T effector cell activation (24). In Sf PM we detected upregulated levels of the crucial Treg induction mediators (32, 33) IL-10 and B7-H1 (PD-L1), which might reflect their attempt to support Treg generation. To gain insight into the capacity of the highly activated PM to reprogram their phenotype, we transferred PM from Sf mice to a non-inflamed PerC and observed that the new environment was sufficient to reshape their immune expression pattern. The expression of key inflammation-inducing molecules in Sf PM downregulated upon their transfer to Wt mice, indicating that these cells are not permanently polarized and that they are able to adapt their activation state in order to re-establish immune homeostasis.

In 2010, Ghosn et al. reported two resident macrophage populations in murine PerC (10). Here, we demonstrate the novel finding that the cellular composition of the peritoneal immune cells is profoundly altered by the disappearance of SPM and accumulation of LPM in the FOXP3-deficient mice. A clear change in the LPM and SPM frequencies was also observed after stimulation with LPS, thioglycolate (10), zymosan, *Trypanosoma cruzi* (13), *Staphylococcus aureus* (15) and in Gata6^−/−^ (34) and *Cebpb*^−/−^ mice (35). However, in contrast to our findings, in these models, LPM population dwindled upon inflammatory challenge whereas the SPM fraction became dominant. Under steady state conditions LPM and SPM share highly similar gene expression profiles (12). SPM have been suggested to represent a resident, short-lived and less mature macrophage population which derives from newly recruited monocytes (10, 34) and serves to replenish LPM under inflammatory conditions (35). Due to the remarkably low amount of SPM in Sf mice, LPM appear to be the central effectors in the inflamed PerC. In comparison to Wt counterparts, Sf LPM displayed high cellular activation as evinced by high apoptotic and autophagic activity as well as increased expression of pro-inflammatory cytokines and surface markers, which was not the case with Sf SPM. In addition, when compared to Sf LPM, Sf SPM displayed significantly disparate expression levels of surface markers involved in cell clearance, migration, cell survival, and regulation of the immune cascade. This finding suggests the distinct origin and function of LPM and SPM during homeostasis which is even more pronounced in the inflammatory conditions. We previously detected elevated myelopoiesis rate and high Ly6C^high^ monocyte count in the bone marrow and peripheral blood of FOXP3-deficient mice (7). Thus, it is feasible that in Treg absence a high demand for replacement of activated and exhausted LPM drives increased monocyte production and recruitment to PerC which, as a result of fast turnover of SPM into LPM, only shortly retain in the SPM state. Nevertheless, events underlying SPM disappearance from the inflamed PerC of Sf mice remain to be resolved.

In conclusion, our data demonstrate a consequential link among Tregs and distribution and functional polarization of PM subsets. A better understanding of the interaction between these cells may not only provide insights into basic mechanisms of immune control, but on the long run may also help to improve current approaches for treating patients with IPEX syndrome and other forms of immune-mediated enteropathies.

## Ethics statement

This study was carried out in accordance with the recommendations of Lower Saxony State Office for Consumer Protection and Food Safety (LAVES). The protocol was approved by the Lower Saxony State Office for Consumer Protection and Food Safety (LAVES).

## Author contributions

JS, AJ, CH, and GH contributed conception and design of the study. JS, AJ, AH, ST, RP, RG, MA, and CH acquired and analyzed experimental data. JS, AJ, RP, CK, CH, and GH interpreted data. JS wrote the first draft of the manuscript. All authors contributed to manuscript discussion and revision, read and approved the submitted version.

### Conflict of interest statement

The authors declare that the research was conducted in the absence of any commercial or financial relationships that could be construed as a potential conflict of interest.
