# Supplementary material for: Absence of Regulatory T Cells Causes Phenotypic and Functional Switch in Murine Peritoneal Macrophages
Source: Front Immunol. 2018 Oct 31;9:2458. doi: 10.3389/fimmu.2018.02458 (PMC6220442; doi:10.3389/fimmu.2018.02458)
Supplement: Supplementary file 1 [file Data_Sheet_1.PDF]

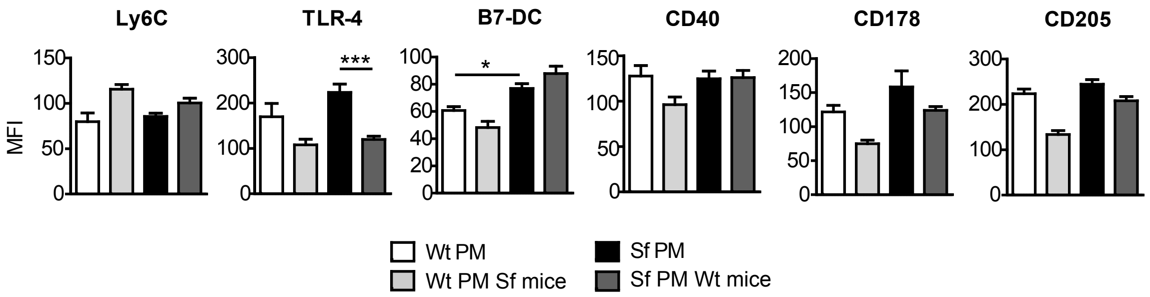

Supplementary Figure S1. Surface marker stainings of the scurfy (Sf) and control (Wt) peritoneal macrophages (PM) from the untreated mice and after their transfer to Wt or Sf peritoneal cavity, respectively, performed by single-cell chipcytometry. Data are depicted as mean fluorescent intensities (MFI) for each surface marker expressed by single cells and shown as bar graphs (n=60 cells per group pooled from more than 8 donor mice). Statistical analyses were performed using one-way ANOVA, * p<0.05, *** p<0.001.
