# Supplementary material for: Absence of Regulatory T Cells Causes Phenotypic and Functional Switch in Murine Peritoneal Macrophages
Source: Front Immunol. 2018 Oct 31;9:2458. doi: 10.3389/fimmu.2018.02458 (PMC6220442; doi:10.3389/fimmu.2018.02458)
Supplement: Supplementary file 2 [file Data_Sheet_2.PDF]

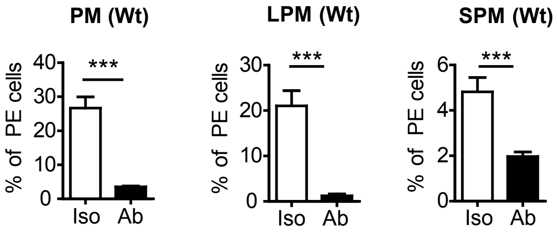

Supplementary Figure S2. Percentages of total peritoneal macrophages (PM), large peritoneal macrophages (LPM) and small peritoneal macrophages (SPM) in peritoneal exudate of wild type (Wt) mice after *in vivo* neutralization of M-CSF with anti-M-CSF specific antibody (Ab) or isotype (Iso) control (n=7-8 mice, data are pooled from two independent experiments). Statistical analysis was performed using unpaired Student's t-test, *** $p<0.001$.
