# Supplementary material for: Absence of Regulatory T Cells Causes Phenotypic and Functional Switch in Murine Peritoneal Macrophages
Source: Front Immunol. 2018 Oct 31;9:2458. doi: 10.3389/fimmu.2018.02458 (PMC6220442; doi:10.3389/fimmu.2018.02458)
Supplement: Supplementary file 3 [file Data_Sheet_3.PDF]

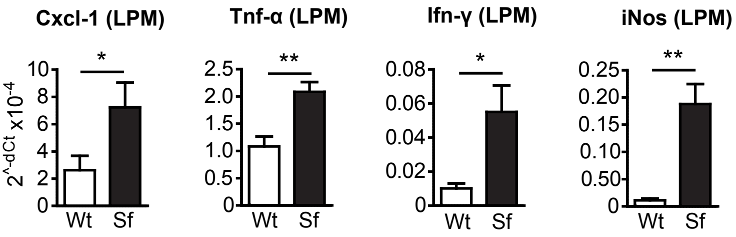

Supplementary Figure S3. Quantitative PCR analysis of gene expression in large peritoneal macrophages (LPM), isolated from scurfy (Sf) and control (Wt) mice (n=4-7 samples per group, each sample contains pooled cells from 3-7 mice). Statistical analyses were performed using unpaired Student's t-test, * $p<0.05$, ** $p<0.01$.
