# Supplementary material for: Absence of Regulatory T Cells Causes Phenotypic and Functional Switch in Murine Peritoneal Macrophages
Source: Front Immunol. 2018 Oct 31;9:2458. doi: 10.3389/fimmu.2018.02458 (PMC6220442; doi:10.3389/fimmu.2018.02458)
Supplement: Supplementary file 4 [file Data_Sheet_4.PDF]

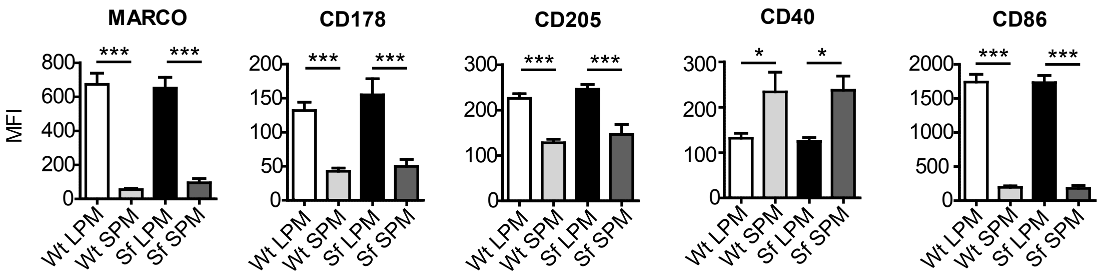

Supplementary Figure S4. Expression of markers involved in immune response in large peritoneal macrophages (LPM) and small peritoneal macrophages (SPM) analysed by single-cell chipcytometry. Data are depicted as mean fluorescent intensities (MFI) for each surface marker expressed by single cells (n=28-62 cells per experimental group, each sample contains pooled cells from more than 8 donor mice). Statistical analyses were performed using one-way ANOVA, * p<0.05, *** p<0.001.
