# Supplementary material for: Absence of Regulatory T Cells Causes Phenotypic and Functional Switch in Murine Peritoneal Macrophages
Source: Front Immunol. 2018 Oct 31;9:2458. doi: 10.3389/fimmu.2018.02458 (PMC6220442; doi:10.3389/fimmu.2018.02458)
Supplement: Supplementary file 6 [file Data_Sheet_6.PDF]

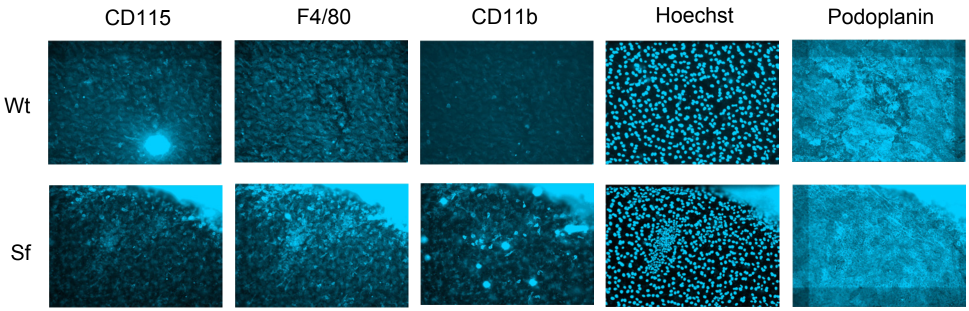

Supplementary Figure S6. Original chipcytometry stainings of the *omentum majus* cryosections obtained from wild type (Wt) and scurfy (Sf) mice. Positive signals from each staining were presented in different colors in Figure 1C.
