# Supplementary material for: Absence of Regulatory T Cells Causes Phenotypic and Functional Switch in Murine Peritoneal Macrophages
Source: Front Immunol. 2018 Oct 31;9:2458. doi: 10.3389/fimmu.2018.02458 (PMC6220442; doi:10.3389/fimmu.2018.02458)
Supplement: Supplementary file 7 [file Data_Sheet_7.PDF]

Epitope	*Fluorophore*	*Clone*	*Manufacturer*	*Epitope*	*Fluorophore*	*Clone*	*Manufacturer*	*Epitope*	*Fluorophore*	*Clone*	*Manufacturer*
CD115	PE-Cy7, PE	AFS98	eBioscience	CD80	PE	16-10A1	BD	MHCII	PE	M5/114.15.2	eBioscience
CD11b	PerCP-Cy5.5, PE	M1/70	BD	CD86	PE	GL1	BD	CD206	PE	C068C2	BioLegend
F4/80	APC, PE	BM8	eBioscience	CD40	PE	3/23	BD	CD47	PE	miap301	BioLegend
CD3	PE	145-2C11	BD	MARCO	PE	579511	R&D	Ly6C	PE	HK1.4	eBioscience
CD19	PE	1D3	BD	B7-DC	PE	TY25	BD	CD178	PE	MFL3	BD
Gr-1	PE	RB6-8C5	BD	CD205	PE	NLDC-145	Miltenyi Biotec	CD45.1	FITC	A20	BioLegend
Siglec-F	PE	E50-2440	BD	CD16/32	PE	93	eBioscience	CD45.1	PE	A20	BD
CD11c	PE	HL3	BD	TLR-2	PE	6C2	eBioscience	CD45.2	APC	104	BioLegend
B7-H1	PE	MIH5	eBioscience	TLR-4	PE	UT41	eBioscience	CD45.2	PE	104	eBioscience
Podoplanin	PE	8.1.1	BioLegend	CD4	FITC	GK1.5	Miltenyi Biotec				

Supplementary Table S1. Antibodies used for flow cytometry, fluorescence-activated cell sorting, and chipcytometry.
