# Supplementary material for: Absence of Regulatory T Cells Causes Phenotypic and Functional Switch in Murine Peritoneal Macrophages
Source: Front Immunol. 2018 Oct 31;9:2458. doi: 10.3389/fimmu.2018.02458 (PMC6220442; doi:10.3389/fimmu.2018.02458)
Supplement: Supplementary file 8 [file Data_Sheet_8.PDF]

Gene	*Assay Nr. (Applied Biosystems)*	*Gene*	*Assay Nr. (Applied Biosystems)*
GATA-6	Mm008012636_m1	TNF-α	Mm00443258_m1
TREM2	Mm04209422_m1	IFN-γ	Mm01168134_m1
Fn-1	Mm01256744_m1	iNOS	Mm00440485_m1
CXCL-1	Mm04207460_m1	IL-4	Mm00445259_m1
CCR2	Mm04207877_m1	IL-10	Mm00439614_m1
Beta-actin	Mm00607939_s1	IL-6	Mm00446190_m1
M-CSF	Mm00432686_m1		

Supplementary Table S2. Primers used for quantitative PCR.
